# Supplementary material for: Development of a BiAD Sensor for Locus-Specific Detection of Cellular Histone Acetylation Dynamics by Fluorescence Microscopy
Source: Genes (Basel). 2025 Apr 10;16(4):444. doi: 10.3390/genes16040444 (PMC12027405; doi:10.3390/genes16040444)
Supplement: Supplementary file 1 [file genes-16-00444-s001.zip › genes-3535847-supplementary.pdf]

Supplementary Figures

Figure S1: Sequence alignment of the BD of BRD9 with the BD1 and BD2 of BRD2.

Figure S2: Testing of the single BRD9 bromodomain for detection of histone acetylation at the *TTC34* locus.

Figure S3: Validation of the efficiency of the TSA treatment.

Figure S4: Compilation of all pairwise p-values of the comparisons of the 2xBRD9-BD BiAD signals shown in Figure 3D.

Figure S1: Sequence alignment of the BD of BRD9 with the BD1 and BD2 of BRD2. BRD2-BD1 Y113, Y186 in BRD2-BD2 and their corresponding residue Y57 in BRD9-BD are highlighted in green. The Y113A mutation in BRD2-BD1 has been shown to disrupt acetyllysine binding.

```
BRD9-BD  : PHGFFAFPVTD------AIAPGYSMIIKHPMDFGTMKDKIVANEYKSVTEFKADFKLMCDNAMTYNRPDTVYY : 107
BRD2-BD1 : WKHQFAWPFRQPVDAVKLGLPDYHKIIKQPMDMGTIKRRLENNYYWAASECMQDFNTMFTNCYIYNKPTDDIV : 163
BRD2-BD2 : KHAAYAWPFYKPVDASALGLHDYHDIIKHPMDLSTVKRKMENRDYRDAQEFAADVRLMFSNCYKYNPPDHDVV : 236
```

Figure S2: Testing of the single BRD9 bromodomain for detection of histone acetylation at the *TTC34* locus. HEK293 cells were transfected with all components of the dual-color BiAD sensor for histone acetylation detection at the *TTC34* target locus with either the wildtype single BRD9BD detector (WT) or a corresponding binding-deficient mutant (Y57A). **A)** Exemplary fluorescence microscopy images showing lack of colocalization of the marker fluorophore (YPet) with a BiAD signal (IFP2.0) for both WT and Y57A detectors. Scale bars are 5 µm and 1 µm for the magnified images. Cell nuclei are indicated by dotted lines. Total number of analyzed cells: WT 10, mutant 10. **B)** Boxplot showing the relative BiAD signals of one experiment. Significance was determined via a two-tailed, unpaired t-test. **C)** P-values for differences in BiAD signal between WT and Y57A for individual experiments using either the single BRD9-BD compared with the corresponding p-values of the double domain detector 2xBRD9-BD taken form Figure 2.

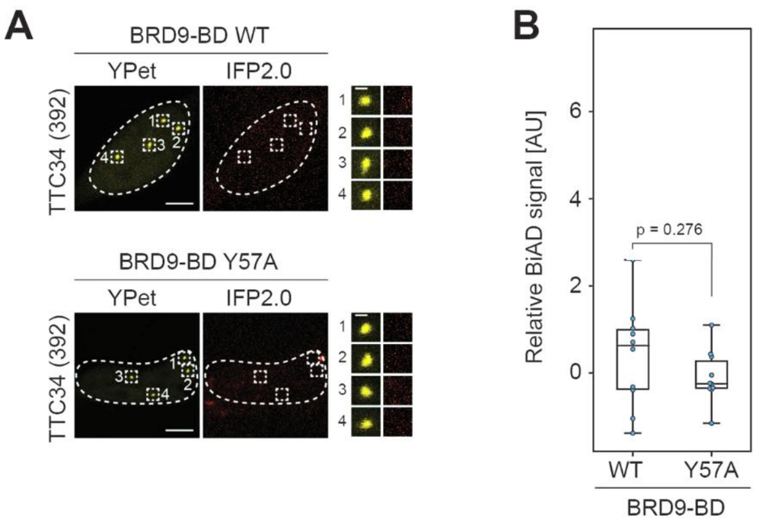

C

	p-value
BRD9-BD	0.276
2xBRD9-BD exp1	0.018
2xBRD9-BD exp2	0.003
2xBRD9-BD exp3	0.030

Figure S3: Validation of the efficiency of the TSA treatment. A) Coomassie BB stained SDS-PAGE gel showing the histone proteins extracted from HEK293 cells with and without TSA treatment. **B)** Western blot of the histone proteins shown in panel A with an α-H4 pan-acetyl Ab showing a strong increase of the histone acetylation signal after TSA treatment. **C)** Same as panel B, but the 6 h TSA sample has been loaded in different dilutions. A semiquantitative analysis of the data revealed that the histone acetylation signal in the 6 h TSA sample is about 10 times stronger than in the DMSO treated cells.

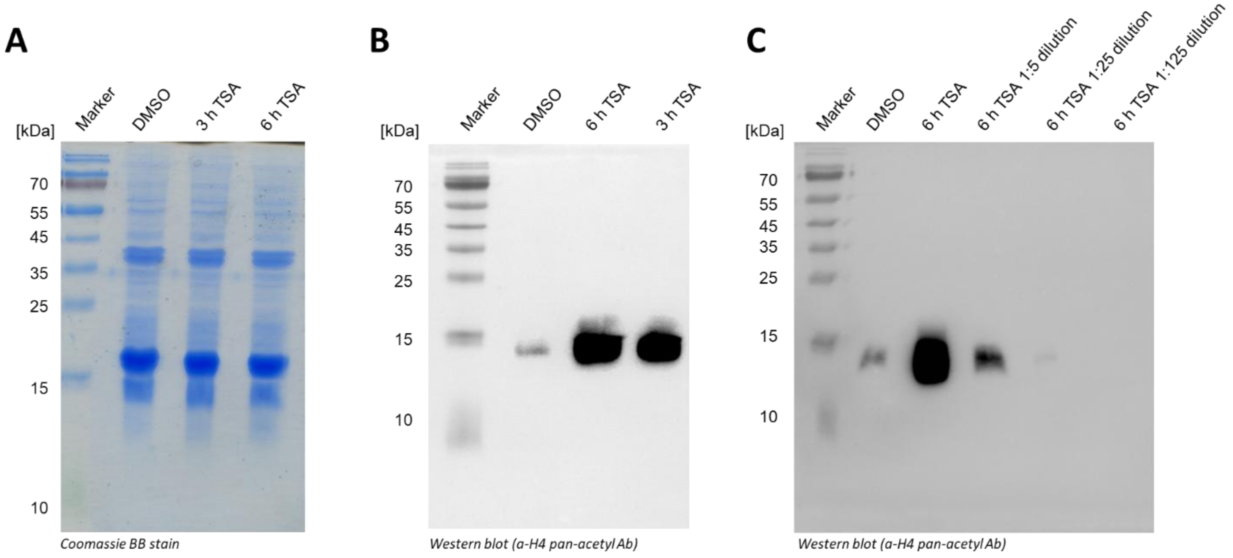

Figure S4: Compilation of all pairwise p-values of the comparisons of the 2xBRD9-BD BiAD signals shown in Figure 3D. The significance of pairwise differences of BiAD signals was determined via a two-tailed, unpaired t-test. Note the highly significant signal change with BiAD sensor containing the WT 2xBRD9-BD when comparing the TSA 6 h sample, as well as the difference between WT and mutant sensors, which do not detect a signal change upon TSA treatment.

**p-value**		**WT**			**Y57A**		
		**DMSO**	**TSA 3 h**	**TSA 6 h**	**DMSO**	**TSA 3 h**	**TSA 6 h**
**WT**	**DMSO**	1.0E+00	8.4E-01	7.5E-05	6.0E-03	7.6E-04	7.9E-03
	**TSA 3 h**	8.4E-01	1.0E+00	9.4E-05	1.8E-02	3.3E-03	2.3E-02
	**TSA 6 h**	7.5E-05	9.4E-05	1.0E+00	2.4E-09	1.2E-09	1.2E-08
**Y57A**	**DMSO**	6.0E-03	1.8E-02	2.4E-09	1.0E+00	4.8E-01	9.6E-01
	**TSA 3 h**	7.6E-04	3.3E-03	1.2E-09	4.8E-01	1.0E+00	4.6E-01
	**TSA 6 h**	7.9E-03	2.3E-02	1.2E-08	9.6E-01	4.6E-01	1.0E+00
